# Supplementary material for: Ultrasonic activation of inert poly(tetrafluoroethylene) enables piezocatalytic generation of reactive oxygen species
Source: Nat Commun. 2021 Jun 9;12:3508. doi: 10.1038/s41467-021-23921-3 (PMC8190189; doi:10.1038/s41467-021-23921-3)
Supplement: Supplementary file 1 — Supplementary Information [file 41467_2021_23921_MOESM1_ESM.pdf]

Supplementary Information

Ultrasonic activation of inert poly(tetrafluoroethylene) enables piezocatalytic generation of reactive oxygen species

Yanfeng Wang, Yeming Xu, Shangshang Dong, Peng Wang, Wei Chen, Zhenda Lu, Deju Ye, Bingcai Pan, Di Wu, Chad D. Vecitis, Guandao Gao*.

*Corresponding author. Email: gaoguandao@nju.edu.cn

This PDF file includes:

Supplementary Methods
Supplementary Notes
Supplementary Figs. 1 to 11
Supplementary Table 1
Supplementary References

Supplementary Methods

Chemicals and Materials

PTFE powders (<d> ~ 1-5 μm) and polyethylene oxide (PEO) (M_w=6×10^5) were supplied by Shanghai Macklin Biochemical Co. Ltd. Polyvinylidene fuoride (PVDF, KYNAR® 761) powders (average 2 μm diamter) were supplied by Arkema. A PTFE membrane (100 μm thick) was purchased from Shenzhen Huasheng Plastic Material Co., Ltd. Polyethylene (PE) powders (<d> ~ 5-500 μm) were purchased from Narui New Material Co., Ltd. Commercial barium titanate ($BaTiO_3$) powders (99.9% trace metal basis, average 100 nm), titanium dioxide (TiO_2) (<d> ~ 10-25 nm), 60 wt% PTFE dispersion were purchased from Aladdin Chemistry Co., Ltd.

5,5-dimethyl-1-pyrolin-*N*-oxide (DMPO), 2,2,6,6-tetramethyl-4-piperidinol (TEMP), and dimethyl sulfoxide (DMSO) were purchased from Sigma-Aldrich (U.S.A). The following chemicals were used as received without further purification from Sinopharm Chemical Reagent Co. Ltd., Shanghai, China: Methyl orange (MO), acid orange 7 (AO7), methylene blue (MB) and 4-chlorophenol (4-CP). All solutions were prepared with high-purity water (18.25 MΩ·cm).

PVDF membranes were fabricated by the traditional phase-inversion method. First, 4.5 g PVDF powders were added into a 30 mL NMP solution under continuous mechanical agitation for 6 h at 60 °C. Next, the solution was cast onto a glass plate by a knife blade with a thickness of 100 μm and immersed into deionized water for coagulation.

PTFE fiber membranes were prepared by electrostatic spinning technique. First, 0.2 g Polyoxyethylene powders were dissolved in 10 mL water under continuous magnetic stirring for 12 h to obtain a 2% homogeneous solution. Next, 6 g PEO solution (2%) and 6.46 g 60% PTFE dispersion and 2 mL H_2O were mixed under magnetic stirring for 12 h. Then, the mixed spinning solution was electrospun onto aluminium foil under an electric field (15 kV), flow rate (0.1 mm/min) and distance between the needle tip and the collector (18 cm). After electrospinning process, the electrospun fiber mats were placed into an oven at 60 ℃ for 2 h and then put in high-temperature oven at 400 ℃ for 10 min to remove polyethylene oxide.

Reactive oxygen species (ROS) analysis during ultrasonic irradiation through porcine tissue

Reactive oxygen species were detected by electron spin resonance (ESR) at ambient temperature: •OH was trapped by DMPO, $^1O_2$ was trapped by TEMP, and $•O_2^-$ was trapped by DMPO/DMSO. The reactions were carried out in 10 mL test tubes. Deionized water (50 mL) containing 1 g/L catalyst (*i.e.*, PTFE, $BaTiO_3$) was irradiated in an ultrasonic cleaner for 1 h in prior to experiment. Then, 0.45 mL of the 1 g/L catalyst (*i.e.*, PTFE, $BaTiO_3$) mixture solution and 50 μL of the 1 M DMPO were employed to detect •OH. Then, 0.375 mL of 1 g/L catalyst (*i.e.*, PTFE, $BaTiO_3$) mixture solution and 50 μL of 1 M DMPO and 75 μL of 15 M DMSO were employed to detect $•O_2^-$. Then, 0.45 mL of 1 g/L PTFE mixture solution and 50 μL of 0.5 M TEMP were employed to detect $^1O_2$. After 5 min of ultrasonic irradiation (1.0 MHz, 2.5 W/cm^2, 10% duty cycle) by an ultrasonic therapy device (US-101L, Japan) through 1 cm thick layer of porcine tissue, the solutions were measured on a Bruker EMX-10/12 ESR spectrometer (Germany).

Disinfection of fungus experiments

A piece of PTFE membrane was tightly attached to the inner wall of the 100 mL beaker. 50 mL of *Candida* suspensions at a concentration of 10^5 CFU/mL was placed in a 100 mL beaker. Subsequently, the beaker containing PTFE membrane and *Candida* was irradiated in the ultrasonic cleaner for 15 min.

Detoxification of cells experiments

A piece of PTFE membrane was tightly attached to the inner wall of the 100 mL beaker. 50 mL of human gastric cancer cell suspensions at a concentration of 10^5 CFU/mL was placed in a 100 mL beaker. Subsequently, the beaker containing PTFE membrane and human gastric cancer cells was irradiated in the ultrasonic cleaner for 5 min.

***E. coli* cell morphology characterization**

A piece of PTFE membrane was tightly attached to the inner wall of the 100 mL beaker. 50 mL of *E. coli* suspensions at a concentration of 10^5 CFU/mL was placed in a 100 mL beaker. Subsequently, the beaker containing PTFE membrane and *E. coli* were irradiated in the ultrasonic cleaner for 15 min. After ultrasound reaction, the *E. coli* suspension was pumped through PES membrane. Then the *E. coli* traped by PES membrane was fixed with 1.0% glutaraldehyde for 1 h. After that, the membrane was dried in an oven at 60 °C and sputtered with gold for SEM (Quanta 250 FEG) imaging.

***Candida* cell morphology characterization**

A piece of PTFE membrane was tightly attached to the inner wall of the 100 mL beaker. 50 mL of *Candida* suspensions at a concentration of 10^5 CFU/mL was placed in a 100 mL beaker. Subsequently, the beaker containing PTFE membrane and *Candida* were irradiated in the ultrasonic cleaner for 15 min. After ultrasound reaction, the *Candida* suspension was pumped through PES membrane. Then the *Candida* traped by PES membrane was fixed with 1.0% glutaraldehyde for 1 h. After that, the membrane was dried in an oven at 60 °C and sputtered with gold for SEM (Quanta 250 FEG) imaging.

Human gastric cancer cell morphology characterization

A piece of PTFE membrane was tightly attached to the inner wall of the 100 mL beaker. 50 mL of human gastric cancer cell suspensions at a concentration of 10^5 CFU/mL was placed in a 100 mL beaker. Subsequently, the beaker containing PTFE membrane and human gastric cancer cells were irradiated in the ultrasonic cleaner for 5 min. After ultrasound reaction, the morphology of human gastric cancer cells was performed by microscope.

Supplementary Notes

Calculation of d_{33} of PTFE and PVDF

The piezoelectric coefficient, d_{33}, is related to PFM amplitude as follows:

$$A = d_{33} V_{ac} Q \qquad (1)$$

where Q is the quality factor, and typically ranges from 10 to 100 in air, V_{ac} is an AC driving voltage applied to the specimen through the conductive AFM cantilever tip, here V_{ac} = 1 V, and A is the measured piezo-response amplitude.

Calculation of a pseudo-first-order kinetic constant

A pseudo-first-order kinetic model was employed to fit the sonocatalytic degradation data using the following equation:

$$-\ln(C_t/C_0) = kt \qquad (2)$$

where C_t and C_0 are the concentration at each interval time and the initial concentration, respectively. k and t are pseudo-first order rate constant and irradiation time.

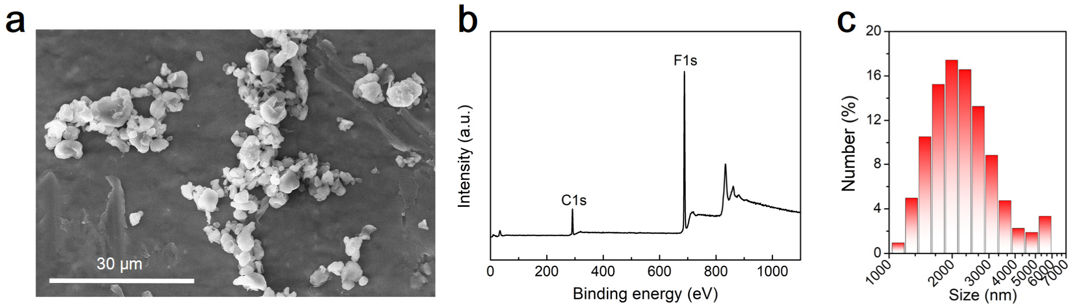

Supplementary Fig. 1 (a) SEM image of PTFE powders. **(b)** XPS spectrum of PTFE powders. **(c)** Particle size distribution of PTFE powders.

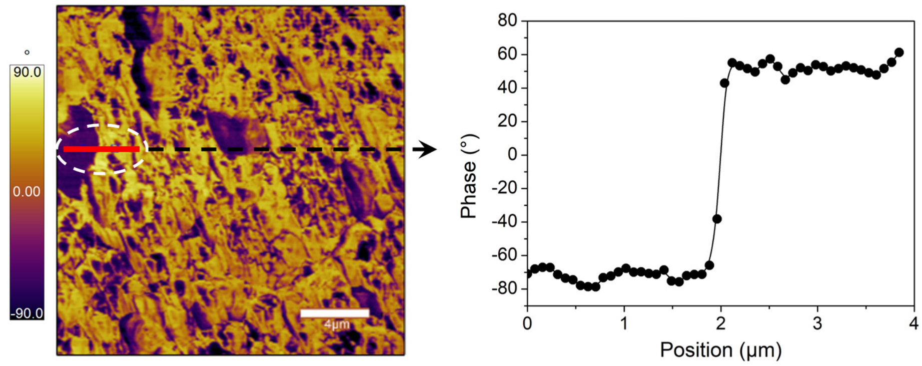

Supplementary Fig. 2 Detailed PFM phase contrast imaging of PTFE membrane activated with ultrasound.

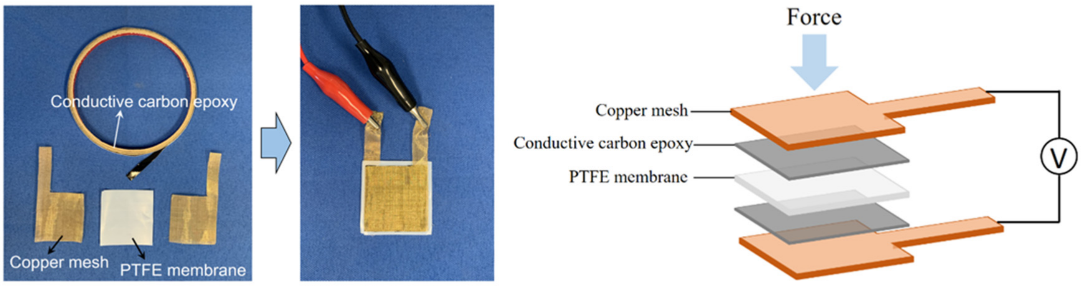

Supplementary Fig. 3 PTFE membrane and Schematic illustration of PTFE membrane device.

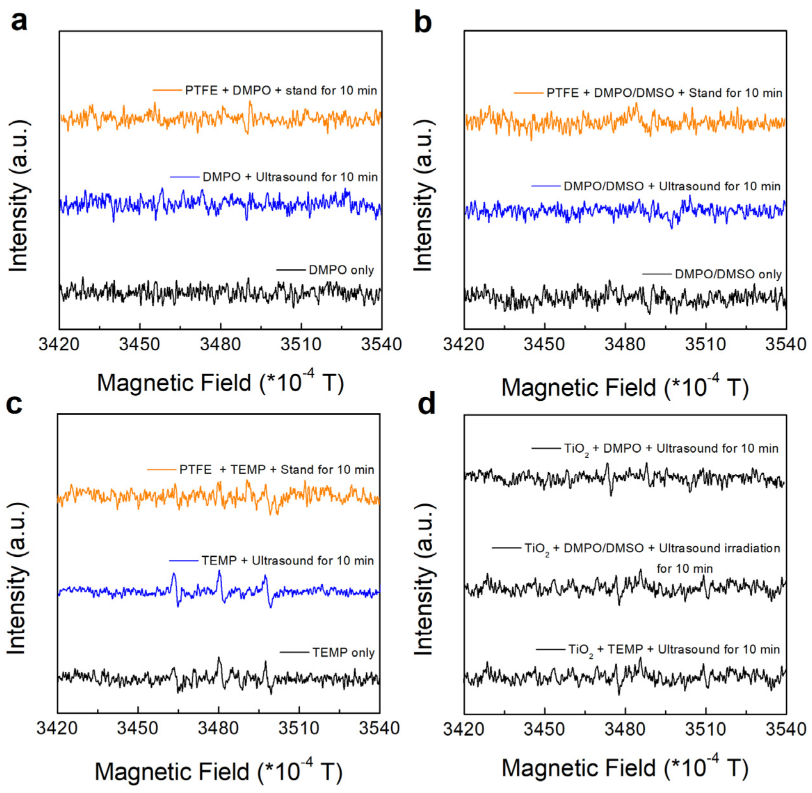

Supplementary Fig. 4 ESR signals of various control experiments. ESR signals for **(a)** DMPO-•OH and DMPO-•H, **(b)** DMPO/DMSO-•O_2^-, **(c)** TEMP-$^1O_2$ in the unpolarized PTFE system. **(d)** ESR signals for DMPO-•OH, DMPO/DMSO-•O_2^- and TEMP-$^1O_2$ in the TiO_2 system. The results of **(a)**, **(b)** and **(c)** showed that there were no obviously •OH, •H, •O_2^- and $^1O_2$ detected if the PTFE was not ultrasonically irradiated. Besides, OH, •H, •O_2^- and $^1O_2$ were not detected with TiO_2 as a catalyst under ultrasonic irradiation **(d)**, which indicated that the activated species existed in PTFE system under ultrasonic irradiation was not caused by ultrasonic cavitation alone.

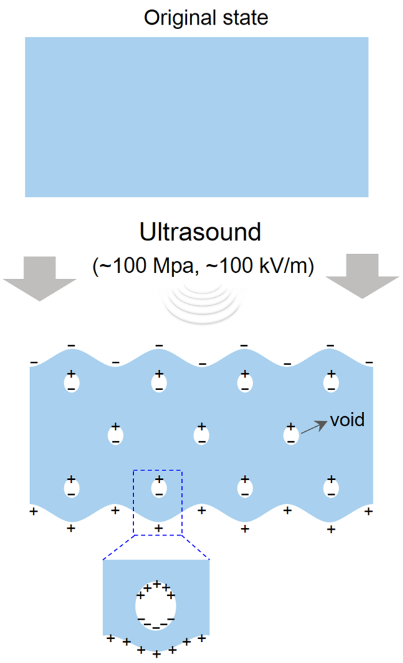

Supplementary Fig. 5 The schematic diagram of PTFE electret formation during ultrasound irradiation

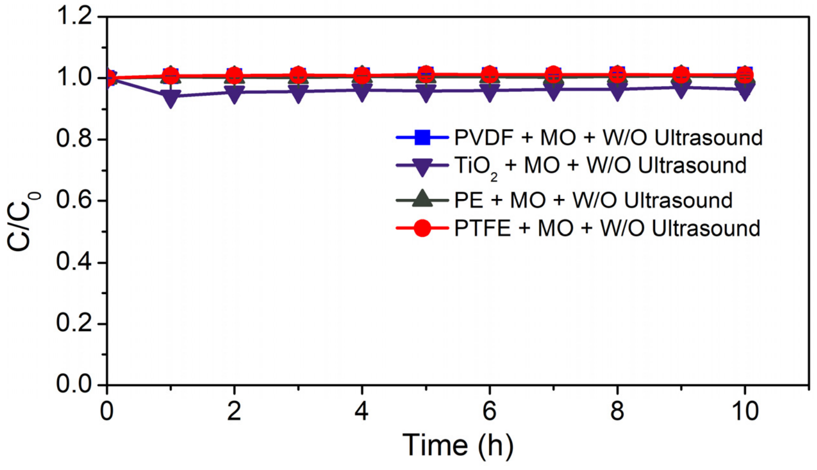

Supplementary Fig. 6 MO removal by PTFE and PVDF, PE, TiO_2 in the absence of ultrasound.

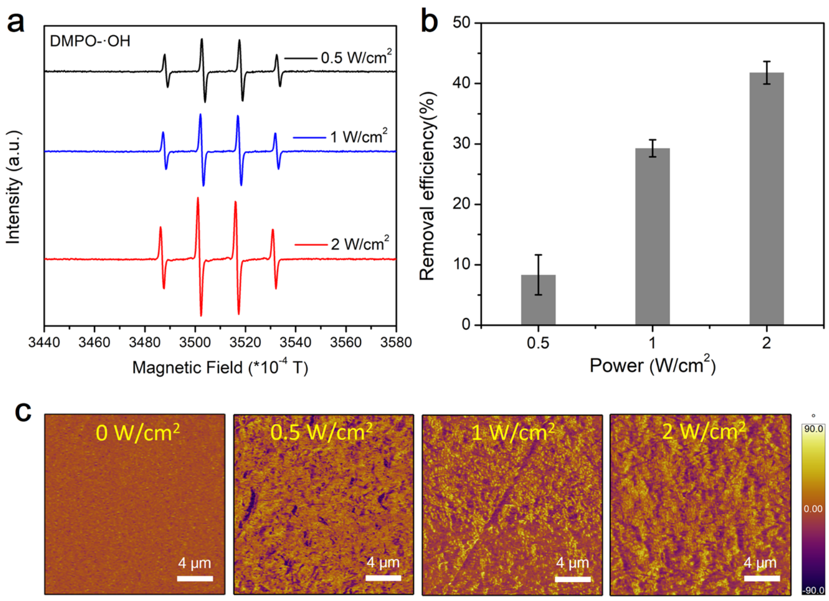

Supplementary Fig. 7 (a) Effect of ultrasound power on •OH generation, **(b)** piezocatalytic degradation of MO ($[PTFE]_0$ = 0.5 g/L, $[MO]_0$=5 mg/L) and **(c)** PFM driven with an ultrasonic therapy device (1.0 MHz, 20% duty cycle, 5 min).

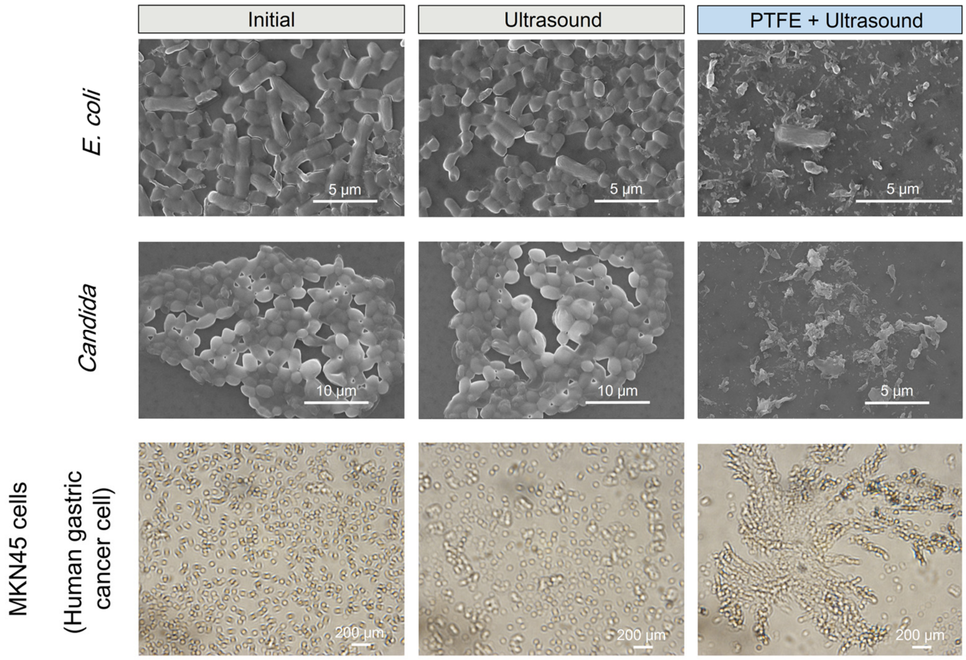

Supplementary Fig. 8 SEM images of *E.coli*, *Candida*, cells before and after ultrasound.

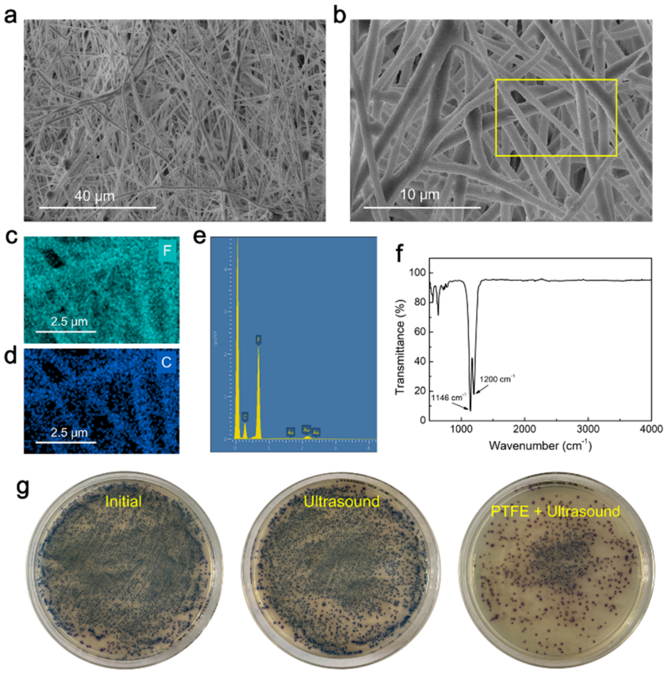

Supplementary Fig. 9 (a, b) SEM image of PTFE fiber membranes prepared by electrospinning and the corresponding EDS mappings of **(c)** C and **(d)** F of the region enclosed by the yellow square. **(e)** The energy dispersive spectrum (EDS) of the yellow square region. **(f)** ATR FTIR of PTFE fiber membranes. **(g)** Photography of *E.coli* colonies on agar culture plates before and after ultrasound with PTFE fiber membranes. The results showed that PTFE fiber membranes could achieve inactivation of about 85.1% of the initial *E.coli*.

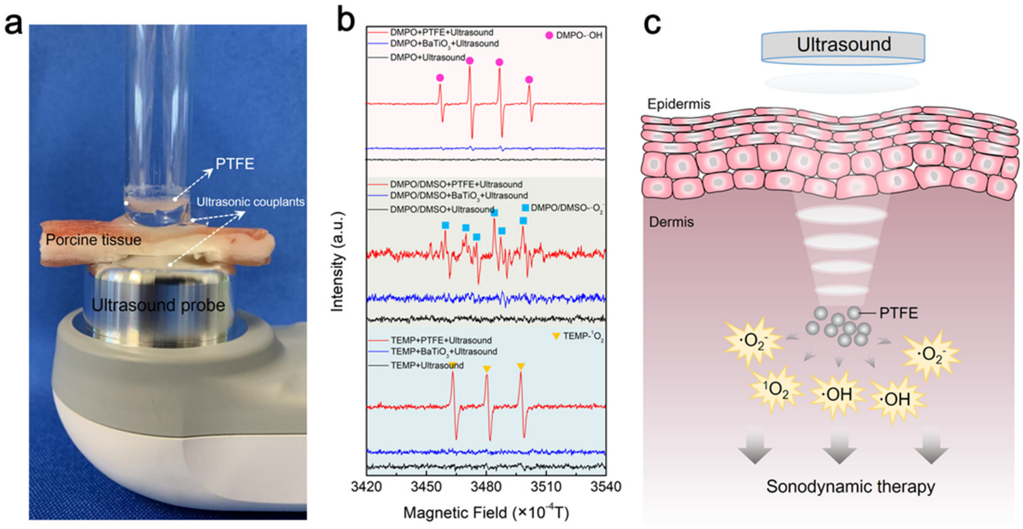

Supplementary Fig. 10 (a) Photograph of ROS generation setup using an ultrasonic therapy device. The thickness of porcine tissue is ~1 cm. **(b)** ESR signals for DMPO-•OH, DMPO/DMSO-•O_2^- and TEMP-$^1O_2$ over PTFE powders under ultrasonic therapy device (1.0 MHz, 2.5 W/cm^2, 10% duty cycle, 5 min), and the simulated tissue solution was covered with ~1 cm thickness of porcine epidermis. **(c)** Schematic illustration of PTFE powders for sonodynamic therapy under ultrasound irradiation.

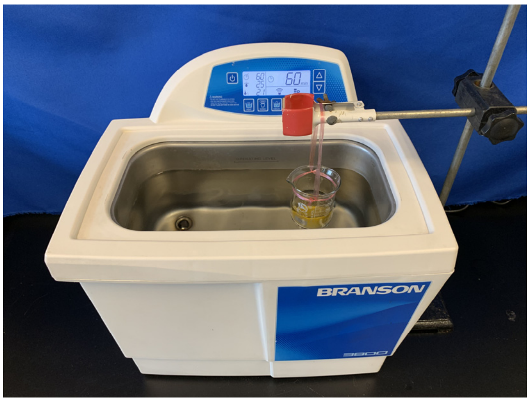

Supplementary Fig. 11 Experimental setup photograph for pollutant degradation and disinfection of bacteria.

Supplementary Table 1. Summary of piezocatalyst kinetic rate constants for organic degradation

Piezocatalyst	Conditions	Pseudo-first order rate constant (min^{-1})	Catalyst normalized rate constant $min^{-1}/(g \cdot L^{-1})$	Ref.
$Pb(Zr_{0.52}Ti_{0.48})O_3$	$[catalyst]_0 = 12.5$ g/L<br>$[AO7]_0 = 30$ μM (10.5 mg/L)	0.0279	0.0022	1
$Bi_4Ti_3O_{12}$	$[catalyst]_0 = 1.33$ g/L<br>$[MO]_0 = 10$ μM (3.27 mg/L)	4.6×10^{-3}	0.0035	2
$BaTiO_3$	$[catalyst]_0 = 1$ g/L<br>$[MO]_0 = 5$ mg/L	0.0150	0.0150	3
$BaTiO_3$	$[catalyst]_0 = 10$ g/L<br>$[AO7]_0 = 57$ μM (20 mg/L)	0.0313	0.0031	4
$BaTiO_3$	$[catalyst]_0 = 9$ g/L<br>$[AO7]_0 = 57$ μM (20 mg/L)	0.0285	0.0032	5
$BaTiO_3$	$[catalyst]_0 = 2$ g/L<br>$[4\text{-}CP]_0 = 25$ mg/L	0.0101	0.0051	6
PTFE	$[catalyst]_0 = 0.25$ g/L<br>$[AO7]_0 = 30$ μM (10.5 mg/L)	**0.0403**	**0.1612**	This study
PTFE	$[catalyst]_0 = 0.25$ g/L<br>$[MO]_0 = 5$ mg/L	**0.0377**	**0.1508**	This study
PTFE	$[catalyst]_0 = 2$ g/L<br>$[4\text{-}CP]_0 = 25$ mg/L	**0.0277**	**0.0139**	This study

Supplementary References

1. H. Lin, et al. Piezoelectrically induced mechano-catalytic effect for degradation of dye wastewater through vibrating $Pb(Zr_{0.52}Ti_{0.48})O_3$ fibers. *Appl. Phys. Lett.* **104**, 162907 (2014).
2. Tu, S. C., Huang, H. W., Zhang, T. R., Zhang, Y. H. Controllable synthesis of multi-responsive ferroelectric layered perovskite-like $Bi_4Ti_3O_{12}$: Photocatalysis and piezoelectric-catalysis and mechanism insight. *Appl. Catal. B-Environ.* **219**, 550-562 (2017).
3. Wu, J., Qin, N., Bao, D. H. Effective enhancement of piezocatalytic activity of $BaTiO_3$ nanowires under ultrasonic vibration. *Nano Energy* **45**, 44-51 (2018).
4. Hong, K.-S., Xu, H. F., Konishi, H., Li, X. C. Piezoelectrochemical effect: A new mechanism for azo dye decolorization in aqueous solution through vibrating piezoelectric microfibers. *J. Phys. Chem. C* **116**, 13045-13051 (2012).
5. Lv, W., et al. Enhancement effect in the piezoelectric degradation of organic pollutants by piezo-Fenton process. *J. Chem. Technol. Biotechnol.*, **92**, 152-156 (2017).
6. Lan, S. Y., et al. Performance and mechanism of piezo-catalytic degradation of 4-Chlorophenol: finding of effective piezo-dechlorination. *Environ. Sci. Technol.* **51**, 6560-6569 (2017).
