# Supplementary material for: Ultrasonic activation of inert poly(tetrafluoroethylene) enables piezocatalytic generation of reactive oxygen species
Source: Nat Commun. 2021 Jun 9;12:3508. doi: 10.1038/s41467-021-23921-3 (PMC8190189; doi:10.1038/s41467-021-23921-3)
Supplement: Supplementary file 3 — Reporting Summary [file 41467_2021_23921_MOESM3_ESM.pdf]

nature research

Corresponding author(s): Guandao Gao
Last updated by author(s): May 7, 2021

Reporting Summary

Nature Research wishes to improve the reproducibility of the work that we publish. This form provides structure for consistency and transparency in reporting. For further information on Nature Research policies, see our Editorial Policies and the Editorial Policy Checklist.

Statistics

For all statistical analyses, confirm that the following items are present in the figure legend, table legend, main text, or Methods section.

n/a	Confirmed	
☐	☒	The exact sample size (*n*) for each experimental group/condition, given as a discrete number and unit of measurement
☐	☒	A statement on whether measurements were taken from distinct samples or whether the same sample was measured repeatedly
☐	☒	The statistical test(s) used AND whether they are one- or two-sided<br>*Only common tests should be described solely by name; describe more complex techniques in the Methods section.*
☒	☐	A description of all covariates tested
☒	☐	A description of any assumptions or corrections, such as tests of normality and adjustment for multiple comparisons
☐	☒	A full description of the statistical parameters including central tendency (e.g. means) or other basic estimates (e.g. regression coefficient) AND variation (e.g. standard deviation) or associated estimates of uncertainty (e.g. confidence intervals)
☒	☐	For null hypothesis testing, the test statistic (e.g. *F*, *t*, *r*) with confidence intervals, effect sizes, degrees of freedom and *P* value noted<br>*Give P values as exact values whenever suitable.*
☒	☐	For Bayesian analysis, information on the choice of priors and Markov chain Monte Carlo settings
☒	☐	For hierarchical and complex designs, identification of the appropriate level for tests and full reporting of outcomes
☒	☐	Estimates of effect sizes (e.g. Cohen's *d*, Pearson's *r*), indicating how they were calculated

Our web collection on statistics for biologists contains articles on many of the points above.

Software and code

Policy information about availability of computer code

Data collection	No software was used
Data analysis	Origin, Microsoft Office

For manuscripts utilizing custom algorithms or software that are central to the research but not yet described in published literature, software must be made available to editors and reviewers. We strongly encourage code deposition in a community repository (e.g. GitHub). See the Nature Research guidelines for submitting code & software for further information.

Data

Policy information about availability of data

All manuscripts must include a data availability statement. This statement should provide the following information, where applicable:

- Accession codes, unique identifiers, or web links for publicly available datasets
- A list of figures that have associated raw data
- A description of any restrictions on data availability

The data supporting the findings of this study are available within the paper and its supplementary information files.

Field-specific reporting

Please select the one below that is the best fit for your research. If you are not sure, read the appropriate sections before making your selection.

☐ Life sciences ☐ Behavioural & social sciences ☒ Ecological, evolutionary & environmental sciences

For a reference copy of the document with all sections, see nature.com/documents/nr-reporting-summary-flat.pdf

Ecological, evolutionary & environmental sciences study design

All studies must disclose on these points even when the disclosure is negative.

Study description	Ultrasound can induce generation of reactive oxygen species by poly(tetrafluoroethylene. This approach was used for environmental pollution control and bacterial therapy.
Research sample	Piezocatalytic ROS generation by poly(tetrafluoroethylene was studied.
Sampling strategy	Poly(tetrafluoroethylene was used to conduct the experiments and polyvinylidene fluoride, polyethylene and TiO2 were used as control samples. The consequent error bars were provided in the experimental results.
Data collection	The phase contrast and amplitude response of PTFE were investigated by piezoresponsive force microscopy (PFM). ROS generation was measured by electron spin resonance (ESR). The voltage signals of the PTFE membrane were recorded with a digital multimeter. Piezocatalytic experiments were conducted in a ultrasound cleaner. Y.F.W. finished experiments and G.D.G. designed experiments. PFM tests were carried out by Y.M.X.
Timing and spatial scale	All the data were recorded within September, 2017 and February, 2021
Data exclusions	No data were excluded from the analyses.
Reproducibility	Reproducibility of experimental findings were checked and it was found that all the data well-reproducible.
Randomization	not applicable to this study
Blinding	Control experiments were performed to establish/support the findings.

Did the study involve field work? ☐ Yes ☒ No

Reporting for specific materials, systems and methods

We require information from authors about some types of materials, experimental systems and methods used in many studies. Here, indicate whether each material, system or method listed is relevant to your study. If you are not sure if a list item applies to your research, read the appropriate section before selecting a response.

Materials & experimental systems

n/a	Involved in the study	
☒	☐	Antibodies
☒	☐	Eukaryotic cell lines
☒	☐	Palaeontology and archaeology
☒	☐	Animals and other organisms
☒	☐	Human research participants
☒	☐	Clinical data
☒	☐	Dual use research of concern

Methods

n/a	Involved in the study	
☒	☐	ChIP-seq
☒	☐	Flow cytometry
☒	☐	MRI-based neuroimaging
